# Magnetic field compensation coil design for magnetoencephalography

**DOI:** 10.1038/s41598-021-01894-z

**Published:** 2021-11-22

**Authors:** Hermann Kutschka, Christian F. Doeller, Jens Haueisen, Burkhard Maess

**Affiliations:** 1 Institute of Biomedical Engineering and Informatics, Technische Universität Ilmenau, Ilmenau, Germany; 2 Max Planck Institute for Human Cognitive and Brain Sciences, Leipzig, Germany; 3 Hans-Berger Department of Neurology, University Hospital Jena, Jena, Germany; 4 Kavli Institute for Systems Neuroscience, Centre for Neural Computation, The Egil and Pauline Braathen and Fred Kavli Centre for Cortical Microcircuits, Jebsen Centre for Alzheimer’s Disease, NTNU, Norwegian University of Science and Technology, Trondheim, Norway; 5 Institute of Psychology, Leipzig University, Leipzig, Germany

**Keywords:** Biomedical engineering, Engineering, Electrical and electronic engineering

## Abstract

While optically pumped magnetometers (OPMs) can be attached to the head of a person and allow for highly sensitive recordings of the human magnetoencephalogram (MEG), they are mostly limited to an operational range of approximately 5 nT. Consequently, even inside a magnetically shielded room (MSR), movements in the remnant magnetic field disable the OPMs. Active suppression of the remnant field utilizing compensation coils is therefore essential. We propose 8 compensation coils on 5 sides of a cube with a side length of approximately 2 m which were optimized for operation inside an MSR. Compared to previously built bi-planar compensation coils, the coils proposed in this report are more complex in geometry and achieved smaller errors for simulated compensation fields. The proposed coils will allow for larger head movements or smaller movement artifacts in future MEG experiments compared to existing coils.

## Introduction

Magnetoencephalography (MEG) acquires magnetic fields produced by neurons, directly and in real-time. Conventional MEG devices utilize superconducting quantum interference devices (SQUIDs) to measure magnetic fields with femtotesla sensitivity. To achieve super conductivity, these extremely sensitive devices are cooled with liquid helium. Hence, the SQUIDs and the helium are housed in a heavy and well insulated container. The housing of SQUIDs renders their arrangement rigid and creates an inhomogeneous distance, on the order of a few centimeters, between sensors and participants’ heads. Recent achievements in optically pumped magnetometry bring a different type of sensor into play for MEG. Zero field optically pumped magnetometers (OPMs) currently achieve a sensitivity level comparable with SQUID systems^1^. In our lab, we use generation 2 QuSpin zero-field magnetometers. The distance between OPM sensors and participants heads is thereby reduced to less than 1 cm and the sensor arrangement is flexible^2^. Due to the smaller distance between sensors and brain sources, the spatial resolution of multi-channel OPM recordings will likely be superior to that of conventional multi-channel SQUID recordings^3,4^. OPM sensor caps or helmets allow participants to move their heads naturally during experiments^2^. It is possible that we will see a shift from fixed cryogenic MEG devices to wearable, motion robust OPM systems in the coming years^5^. This would open up the possibility of expanding MEG research to new participant groups, like infants or specific patient groups, and to new paradigms where head movements are necessary or encouraged^6,7^.

A limitation of current high sensitivity zero field OPMs is their small operational range. For example, the OPMs of our lab have an operational range of approximately $\pm 5\,{\mathrm{nT}}$. Due to the small operational range it is required that OPMs are operated inside a magnetically shielded room (MSR), to suppress the static earth magnetic field at the sensor positions. Inside an MSR each sensor can generate its own magnetic field by means of internal Helmholtz coils, to meet its operational range around absolute zero field. However, this internal field of each sensor is initially fixed before switching to its sensitive measurement mode. Small head movements in the range of a few millimeters in the remnant magnetic field inside an MSR can already disable the OPMs^2^. To address this head movement problem, compensation coils were built to actively suppress dominant field components of the remnant magnetic field in a volume for all OPMs simultaneously inside an MSR^8–10^. We describe an enhancement of previously built compensation coils. In the following, shielding refers to a passive field reduction by means of deflecting materials, whereas compensation refers to active field suppression by means of electric current driven coils. Large, high quality compensation coils for OPM recordings have previously been designed as bi-planar coils^2,8,10^. Bi-planar coils can reduce the remnant magnetic field sufficiently to even allow OPM recordings during head movement^8^. In a second report^10^, a simplified bi-planar system was designed and the effects of magnetic shielding on magnetic field compensation was investigated. It was found, that the magnetic fields of their coils were considerably effected by the presence of their MSR. This effect did not equally impact the field suppression performance of their coils, because coil interactions are modeled and taken into account during field suppression. In practice, their coils showed good field suppression performance.

In contrast to^8,10^, we propose a coil design that is optimized for operation inside a magnetically shielded environment. To accomplish this we use a direct stream function discretization. With a more complex geometry, we aim at a better compensation in larger target volumes at the expense of a heavier and more elaborate construction compared to^8,10^. Direct stream function discretization provides more freedom in coil geometry and shield modeling. For example, contrary to^8,10^, symmetry is not a requirement. We aimed at compensation errors of $\le 1\%$ inside a sphere with diameter 0.6 m in simulations where magnetic shielding was taken into account. Consequently, we require accurate modeling of shielding effects with errors clearly below our compensation errors.

## Coil design and evaluation

### Preface

The starting point of our coil development was the bi-planar design described in^8^. We identified two potential enhancements, a more surrounding coil geometry and incorporation of the effect of an MSR on the magnetic field in coil optimization. For the geometry enhancement, we considered a cubic design because it allows simple construction. We decided to remove the front face of the cube to provide an easy entrance and exit and to avoid a locked in atmosphere for participants. In favor of a simple construction, we also decided to avoid coil wires over the complete lengths of the edges.

For the incorporation of magnetic shielding we followed up on a method described in^10^ and implemented a method of images. Reference^10^ did not incorporate magnetic shielding in their coil design. We implemented a different magnetic field modeling approach compared to^8,10^ to model our coils. In the following sections, the details of designing and evaluating our compensation coils are described. The term compensation coil refers to one wire with one input and one output contact for electric current.

Our design methods were based on a magnetostatic approximation and we estimated its accuracy in our application. We defined a surface stream function discretization to express the magnetic field in a target grid as a matrix product. The shielding effects were taken into account by virtually mirroring our mesh on the MSR walls. Our coils were constrained to surfaces which were defined on five-sided cuboid meshes with a total side length of approximately 2 m. Our target volume for compensation was a sphere with diameter 0.6 m in the center of the cuboid. Optimization was computed by a regularized least squares regression of the stream function for a specified target field in a grid inside our target volume. Wiring paths of each coil were computed as contour lines of the optimized stream function. From the wiring paths, errors were computed against the target fields.

### Magnetostatic approximation

We were aiming at manufacturing coils from enameled copper wire with diameter $1\,{\mathrm{mm}}$, similar to^8,10^. The magnetic field components, that we were suppressing contain only frequencies below 100 Hz. For this frequency range, we simulated our fields in a magnetostatic approximation

1 $$\begin{aligned} \nabla \times \vec {B} = \mu _0 \vec {J}\,, \end{aligned}$$

where $\vec {B}$ is the magnetic flux density and $\vec {J}$ is a quasi-stationary current density. For a time-dependent current density $\vec {J}\left( t\right)$ in copper wires, we derived an approximation error based on

2 $$\begin{aligned} \nabla \times \vec {B} = \mu _0\left( \vec {J}\left( t\right) +\varepsilon _0\frac{\partial \vec {E}}{\partial t}\right) \end{aligned}$$

where $\vec {E}$ is the electric field. We assumed the electric field amplitude was maximal between two wire segments of opposing current direction close to the current supply of a coil loop, where the two segments are very close to each other. At the center between these segments, we estimate the electric field amplitude as

3 $$\begin{aligned} \Vert \vec {E}\left( t\right) \Vert = \left\Vert \nabla \cdot \left( \rho \cdot \ell + L \cdot A \frac{\partial }{\partial t} \right) \right\Vert \cdot \Vert \vec {J}\left( t\right) \Vert \,, \end{aligned}$$

where $\rho = 1.72\times 10^{-8}\,\Omega \,{\mathrm{m}}$ is the resistivity of copper at $20\,^{\circ }{\text{C}}$, $\ell$ is the wire length, *A* its cross section and *L* is the coil inductance. In our coils, wire length was $\ell < 1 \times 10^{4}\,{\mathrm{m}}$ and inductance was $L < 1 \times 10^{-1}\,{\mathrm{H}}$. Our copper wire cross section was $A \approx 1 \times 10^{-6}\,{\text {m}}^{2}$. For a harmonic current density with a frequency of 100 Hz we set $\frac{\partial }{\partial t} = 2\pi 100$ Hz. Finally, we estimated a worst-case gradient $\nabla$ of $1 \times 10^{5}/{\text {m}}$ for two wires with only a coating of $1 \times 10^{-5}\,{\mathrm{m}}$ between them. The relative magnetostatic approximation error $\delta _{\mathrm{stat}}$ resulted:

4 $$\begin{aligned} \delta _{\mathrm{stat}} = \varepsilon _0 \frac{\partial }{\partial t} \frac{\Vert \vec {E}\left( t\right) \Vert }{\Vert \vec {J} \left( t\right) \Vert } = \varepsilon _0 \frac{\partial }{\partial t} \nabla \cdot \left( \rho \cdot \ell + L \cdot A \frac{\partial }{\partial t} \right) \approx 1.3 \times 10^{-7}. \end{aligned}$$

Compared to our compensation error goal of 1%, $\delta _{\mathrm{stat}}$ is small. Hence, the magnetostatic approximation was sufficiently accurate for our application.

### Stream function solution

We planned to put our wires inside wooden boards where each coil is constrained to a given surface. This idea follows^8,10^, who glued the wire to their boards. We planned to increase accuracy in this manufacturing step by milling traces with a computer numerical control and putting the wire inside. Wire paths for coils constrained to a surface and in the magnetostatic regime can be expressed as contour lines of a scalar stream function. We derive this relation in the following paragraph.

For a current density $\vec {J}$ in magnetostatics, the continuity equation $\nabla \vec {J} = 0$ holds. In the *xy*-plane (for simplicity) this is

5 $$\begin{aligned} \frac{\partial j_x}{\partial x} + \frac{\partial j_y}{\partial y} = 0. \end{aligned}$$

A solution of this partial differential equation (5) follows from symmetry of second derivatives as $j_x = \frac{\partial S}{\partial y}$ and $j_y = -\frac{\partial S}{\partial x}$, where *S* is a scalar function in the *xy*-plane. This can be expressed as

6 $$\begin{aligned} \vec {J} = \nabla \times (S \vec {e}_z) = \nabla S \times \vec {e}_z\,, \end{aligned}$$

which defines a scalar stream function *S*^11^. This stream function concept can be generalized to arbitrary surfaces, where $\nabla$ is replaced by the tangential gradient operator on the surface and $\vec {e}_z$ is replaced by the unit surface normal vector^12^. Stream lines of the surface current density $\vec {J}$ are, by definition, parallel to $\nabla S \times \vec {e}_z$ and hence perpendicular to $\nabla S$. That means, contour lines of *S* are stream lines of $\vec {J}$^12^. Since wire paths are stream lines of a current density, contour lines of *S* defined our coil wire paths.

We used a quadrilateral mesh to discretize stream function surfaces. For each element, the stream function was described by a bilinear function which was determined by the values at the four vertices. However, the continuity equation for current density holds for any twice continuously differentiable function *S*. Considering a standard square with side length $l=1$ m in a Cartesian coordinate system (*x*, *y*, *z*). For $z=0$ and $\left( x, y\right) \in \left[ 0, l\right] \times \left[ 0, l\right]$ and a stream function $S \propto xy$, the magnetic field can be expressed as (Biot-Savart):

7 $$\begin{aligned} \vec {B}&= \lim _{a \rightarrow 0} \frac{\mu _0}{4\pi a} \iiint _{0\, 0\, 0}^{a\, l\, l}{\frac{\left( \nabla^{\prime} \times S^{\prime} \vec {e}_z\right) \times \begin{pmatrix} x - x^{\prime}\\ y - y^{\prime}\\ z \end{pmatrix} {\mathrm{d}}V^{\prime} }{\sqrt{(x - x^{\prime})^2 + (y - y^{\prime})^2 + z^2}^{3}}} \end{aligned}$$

8 $$\begin{aligned}{} & \propto \iint _{0\, 0}^{l\, l}\frac{\begin{pmatrix} -y^{\prime}z\\ -x^{\prime}z\\ x^{\prime} \left( y - y^{\prime}\right) + y^{\prime} \left( x - x^{\prime}\right) \end{pmatrix} {\mathrm{d}}x^{\prime} {\mathrm{d}}y^{\prime}}{\sqrt{(x - x^{\prime})^2 +(y - y^{\prime})^2 + z^2}^{3}} \,, \end{aligned}$$

where *a* is the thickness of the volume on the *z* axis, which approaches zero, ${\mathrm{d}}V^{\prime} = {\mathrm{d}}x^{\prime} {\mathrm{d}}y^{\prime} {\mathrm{d}}z^{\prime}$ and is the volume element, $S^{\prime}$ is the stream function in that volume, $\nabla ^{\prime}$ operates on $x^{\prime}, y^{\prime}$. Although all integrals in (8) can be expressed in closed-form, we faced numerical problems in their expressions. We decided to use a $4\times 4$ point Gauss-Legendre quadrature rule for these integrals instead. At a target point $\vec {r}$ the magnetic field was computed for each single vertex of our quadrilateral mesh with a stream function value of $1\,{\text {A}}/{\text{m}}$ at that vertex and zero at all other vertices. This allowed us to compute the magnetic field at *M* target points $\vec {r}_1 \dots \vec {r}_M$ as a matrix product of a matrix $\varvec{B}$, denoted as a forward matrix, containing the solutions of (8) and a vector $\varvec{s}$ containing the stream function values of all *N* vertices:

9 $$\begin{aligned} \begin{bmatrix} b_x\left( \vec {r}_1\right) \\ \vdots \\ b_z\left( \vec {r}_M\right) \end{bmatrix}&= \begin{bmatrix} b_{x1}\left( \vec {r}_1\right) &{} \dots &{} b_{xN}\left( \vec {r}_1\right) \\ \vdots &{} \ddots &{} \vdots \\ b_{z1}\left( \vec {r}_M\right) &{} \dots &{} b_{zN}\left( \vec {r}_M\right) \end{bmatrix} \cdot \begin{bmatrix} s_1\\ \vdots \\ s_{N} \end{bmatrix} \nonumber \\&= \varvec{B} \cdot \varvec{s}. \end{aligned}$$

**Figure 1:**
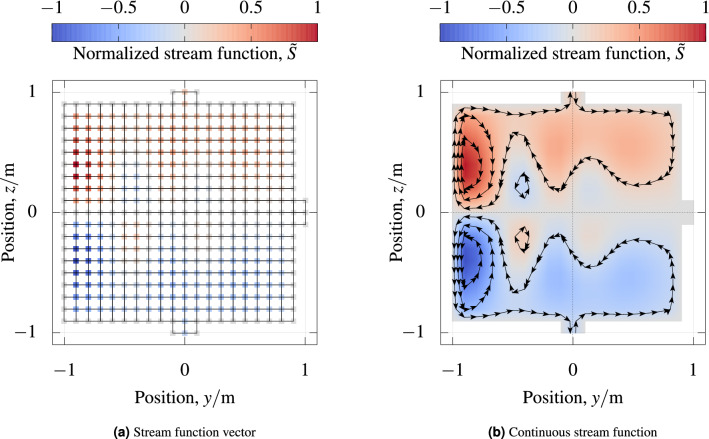
*Stream function* (**a**) Stream function vector of coil $C_{z,\,{\text {hom}}}$ depicted on its mesh vertices. From the cube, only the face with the minimal *x* coordinate is shown. For this face, the same original mesh as in Fig. 2 is depicted with black lines. The mesh elements that stick out at the top, bottom, and right connect this mesh with the adjacent cube faces. In (**b**) the continuous stream function is depicted according to its bilinear definition from the stream function vector in (**a**).

A part of an example stream function vector is depicted in Fig. 1.

### Geometry and mesh

Constraining surfaces of our coils were derived from a base cube surface of $2\,{\mathrm{m}}$ side length. One face of the cube was removed for coil surfaces, specifically the front where participants can enter the cube. This front side faced the door of the MSR. The remaining surface, with 5 faces, was subdivided in $20 \times 20$ square elements on each face of the cube. Elements at the 8 edges of this mesh were removed in favor of a simpler construction. Only the 4 center elements of those edges were kept allowing the current to go from one face to another. In Fig. 3 our final mesh is depicted inside and mirrored on the MSR walls. The dimensions of our MSR were $3.002\,{\mathrm{m}} \times 4.002\,{\mathrm{m}} \times 2.452$ m and our mesh was centered inside on the *x* and *z* axes and shifted off-center 0.65 m negatively on the *y* axis (to the front). We planned to arrange 8 coils in our 5 boards. Practically, it is not possible to lay 8 coils on a single depth in the boards. We decided to lay 4 coils in the inside and 4 in the outside of our boards and to allocate one depth for 2 coils in our boards. Hence, we had 4 depths $-7.5$ mm, $-4.5$ mm, 4.5 mm and 7.5 mm for our coils around the base cube of side length 2 m. The different depths were realized by scaling the base cube side lengths from 2 to 1.9925 m, 1.9955 m, 2.0045 m and 2.0075 m. This is implemented in according shifts of the base mesh and results in 4 slightly different meshes for our computation. The mesh for one coil consisted of 1905 vertices and 1724 square elements. Columns of the forward matrix were only defined for the 1537 non-boundary vertices. This is because stream function contour lines must not cross the boundary and hence the stream function must be constant at a boundary.

**Figure 2:**
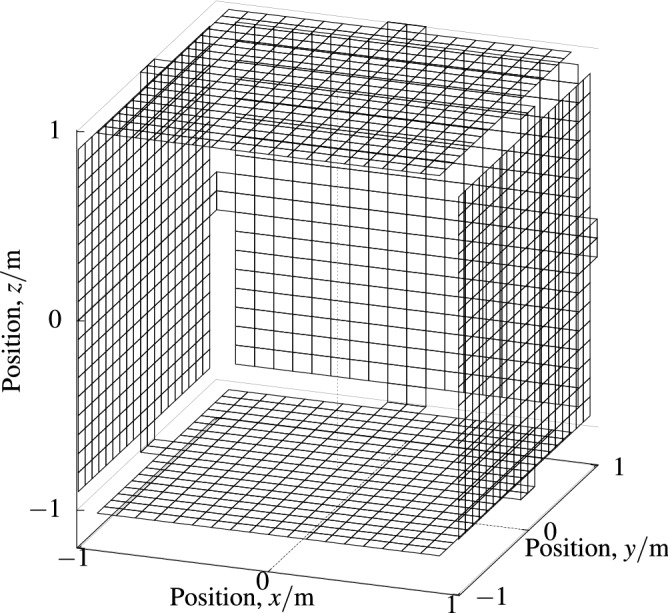
*Surface mesh* 3D view of one of our 4 quadrilateral surface meshes where coils are constrained to.

### Magnetic shield modeling

The magnetic field is compensated for inside an MSR. For differentiation from coil geometries, the sides of our MSR are referred to as walls. The walls of the MSR consisted of two sheets of $\mu$-metal to shield the static and low frequency components of the magnetic field (mainly the earth’s magnetic field). Relative permeability $\mu _r$ of $\mu$-metal is high, typically $> 8 \times 10^{4}$, while that of air is approximately 1. The effect of an abrupt change in permeability from air to $\mu$-metal on the magnetic field can be described by the following two differential equations

10 $$\begin{aligned} \nabla \vec {B}&= 0 \end{aligned}$$

11 $$\begin{aligned} \nabla \times \vec {H}&= \vec {J}_f = \vec {0} \end{aligned}$$

and the material equation $\vec {B} = \mu _r \mu _0 \vec {H}$, where $\vec {J}_f$ is the free current density, which is zero in $\mu$-metal and air. According to Gauss’s law in (10), there is no discontinuity in the normal component of magnetic flux density $\vec {B}$ across the boundary between air and $\mu$-metal. There is also no discontinuity in the tangential component of $\vec {H}$ across this boundary according to Ampere’s law in (11). Both differential equations, together with the material equation, require an abrupt change of magnetic field direction across the boundary of air and $\mu$-metal. The magnetic field enters the $\mu$-metal boundary nearly perpendicular from the air side and is nearly tangential to that boundary inside the $\mu$-metal^13^. To simulate the effect of $\mu$-metal on the magnetic field at the air side, a method of images can be applied^8^. That is, currents inside the MSR are mirrored on the MSR walls. The sensitivity of this method to the value of $\mu _r$ is small here since the simulation errors scale with $\mu _r^{-1}$, which is negligible for $\mu$-metal^10^.

Since $\mu$-metal is mounted on all 6 walls of the MSR, mirroring of a current across one wall requires mirroring of the mirrored current across the other walls and so on. Theoretically, the mirroring proceeds to infinity for an accurate simulation. Practically, it can be truncated at a certain level of accuracy because the distance *r* of the mirrored currents to the target volume increases with each level and hence the effect on the magnetic field decreases with $r^{-1}$. To determine the level of truncation, we introduced a set of indices ${\mathscr {L}}$ for a level of mirroring. Elements of ${\mathscr {L}}$ are triples of integer numbers representing the number of sequential mirroring along one of the three spatial axes. The sign of a number indicated the direction on the axis. For example, the element $\left( 0, 1, -2\right)$ represented a mirroring across the wall in positive *y* direction followed by a mirroring across the wall in positive *z* direction, and a second mirroring across the wall in negative *z* direction. A set of mirrorings, up to a total order of *P*, was defined as

12 $$\begin{aligned} {\mathscr {L}}_P = \left\{ \left( x, y, z\right) \in {\mathbb {Z}^3}: 0 < |x |+ |y |+ |z |\le P \right\} . \end{aligned}$$

The definition of the forward matrix $\varvec{B}$ in (9) is extended to the mirroring and is expressed as:

13 $$\begin{aligned} \varvec{B}\left( {\mathscr {L}}_n\right) = \sum _{\left( k, l, m\right) \in {\mathscr {L}}_n}{\varvec{B}\left( k, l, m\right) }\,, \end{aligned}$$

where $\left( k, l, m\right)$ are the mirroring indices on $\left( x, y, z\right)$ axes for the stream function mesh.

**Figure 3:**
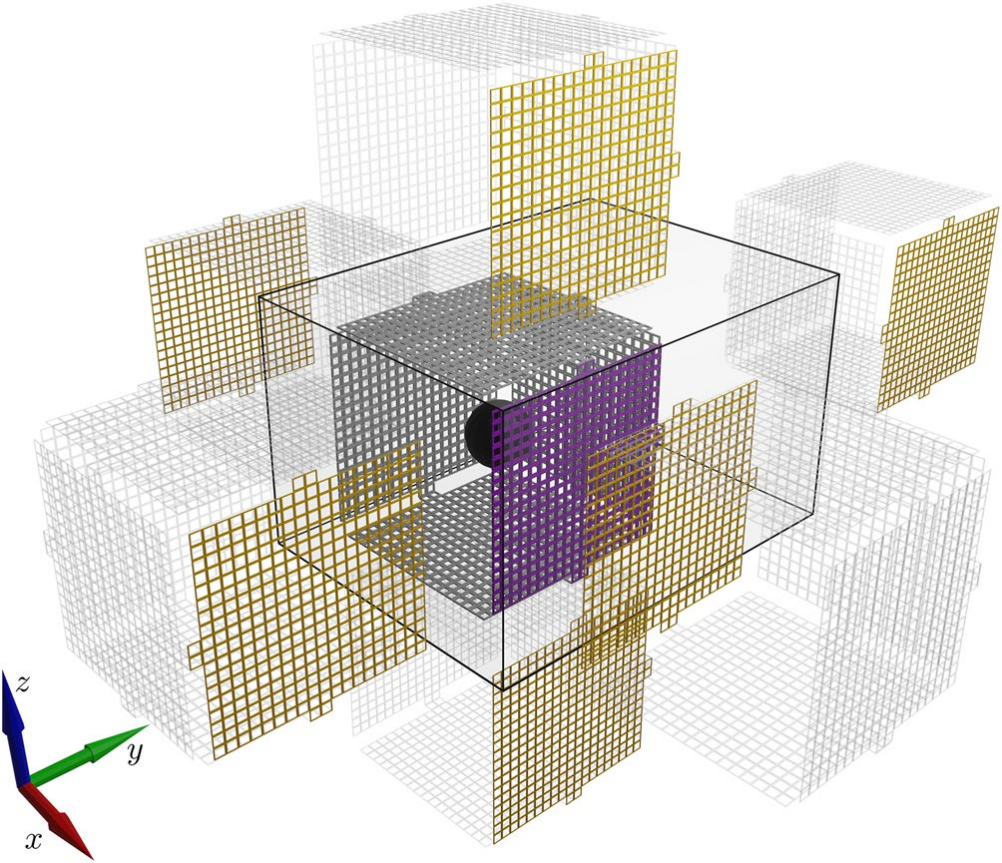
*Method of images* 3D rendering of a level 1 mirroring of a surface mesh. The original mesh is gray colored with one side highlighted in violet. Mirrorings of this mesh are rendered transparent but mirrorings of the highlighted side are again highlighted in orange. The edges of the MSR are rendered in black. In the center, inside the original mesh, a black sphere with diameter 0.6 m is rendered, it depicts the target volume of our coil optimization. 3D models of different mirroring levels are depicted in the supplementary material.

Table 1 lists the total number of mirrorings for different levels *n* and the number of additional mirroring compared to the preceding level $n-1$ in columns 2 and 3 respectively. That is, column 3 reveals the increase in total mirrorings from one level to the next. Figure 3 depicts level 1 mirroring of a surface mesh on the MSR.

**Table 1:** For increasing mirroring levels *n*: the number of mirrorings on MSR walls and the difference in that number to the preceding level.

Level *n*	Number of mirrorings $|{\mathscr {L}}_n |$	Number of additional mirrorings $|{\mathscr {L}}_n \setminus {\mathscr {L}}_{n-1} |$
1	6	6
2	24	18
3	62	38
4	128	66
$\cdot$	$\cdot$	$\cdot$
7	568	192

### Target field

**Figure 4:**
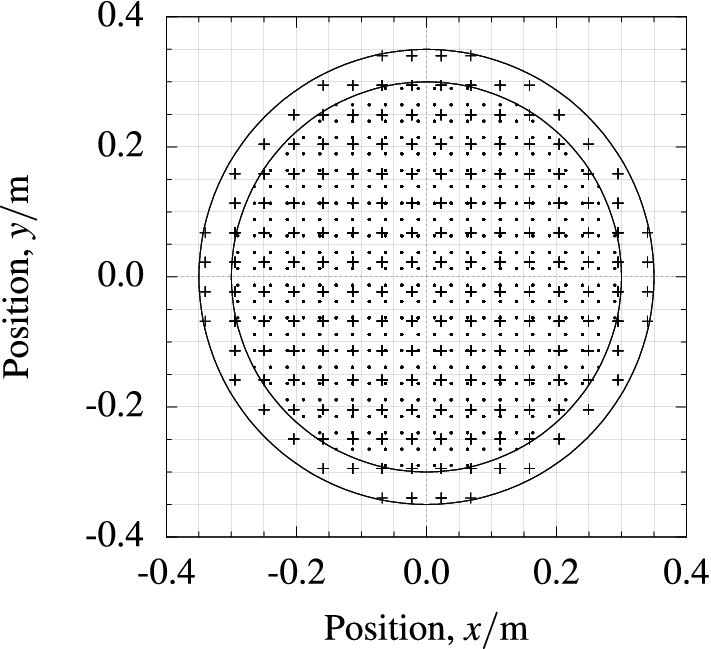
*Target grids* Target points ${\mathscr {T}}_{\mathrm{opt}}$ are marked with $+$-crosses and enclosed inside a circle with diameter 0.7 m. Validation points ${\mathscr {T}}_{\mathrm{val}}$ are marked with dots and enclosed inside a circle of 0.6 m diameter. Point sets are depicted in orthographic view from the *z* axis.

An equidistant grid of points was defined within a centered cube and all grid points within the centered sphere of diameter *d* defined the target set. This method defined a volumetric equidistant sampling of a spherical volume. For stream function optimization, a target grid ${\mathscr {T}}_{\mathrm{opt}}$ was defined with a diamater of 0.7 m and sampling distance of 0.045 m. Similarly for validation ${\mathscr {T}}_{\mathrm{val}}$ was defined with 0.6 m and sampling distance of 0.025 m. ${\mathscr {T}}_{\mathrm{opt}}$ and ${\mathscr {T}}_{\mathrm{val}}$ were disjoint sets in order to avoid inverse crime. ${\mathscr {T}}_{\mathrm{val}}$ was more fine grained than ${\mathscr {T}}_{\mathrm{opt}}$ to validate the interpolation of our optimization. The number of grid points in the two sets were $|{\mathscr {T}}_{\mathrm{opt}} |= 1904$, $|{\mathscr {T}}_{\mathrm{val}} |= 7088$. Cross sections of the target grids are depicted in Fig. 4.

The actual target fields for optimization were defined for all $\vec {r} \in {\mathscr {T}}_{\mathrm{opt}} = \left\{ \vec {r}_1 \dots \vec {r}_M\right\}$ as

14 $$\begin{aligned} \vec {B}_{x,\,{\mathrm{hom}}} \left( \vec {r}\right)&\propto \left( 1, 0, 0\right) \end{aligned}$$

15 $$\begin{aligned} \vec {B}_{y,\,{\mathrm{hom}}} \left( \vec {r}\right)&\propto \left( 0, 1, 0\right) \end{aligned}$$

16 $$\begin{aligned} \vec {B}_{z,\,{\mathrm{hom}}} \left( \vec {r}\right)&\propto \left( 0, 0, 1\right) \end{aligned}$$

17 $$\begin{aligned} \vec {B}_{x,\,\nabla _y} \left( \vec {r}\right)&\propto \left( y, x, 0\right) \end{aligned}$$

18 $$\begin{aligned} \vec {B}_{x,\,\nabla _x} \left( \vec {r}\right)&\propto \left( x, -y/2, -z/2\right) \end{aligned}$$

19 $$\begin{aligned} \vec {B}_{z,\,\nabla _z} \left( \vec {r}\right)&\propto \left( -x/2, -y/2, z\right) \end{aligned}$$

20 $$\begin{aligned} \vec {B}_{x,\,\nabla _z} \left( \vec {r}\right)&\propto \left( z, 0, x\right) \end{aligned}$$

21 $$\begin{aligned} \vec {B}_{z,\,\nabla _y} \left( \vec {r}\right)&\propto \left( 0, z, y\right) . \end{aligned}$$

For matrix expressions, target field vectors were defined as $\varvec{b}_{\mathrm{target}} = \left( \vec {B}_{\mathrm{target}} \left( \vec {r}_1\right) , \dots , \vec {B}_{\mathrm{target}} \left( \vec {r}_M\right) \right) ^{\mathsf {T}}$. The compensation coils were named according to their target fields as $C_{x,\,{\text {hom}}} \dots C_{z,\,\nabla _y}$.

### Stream function optimization and wire paths

As defined in (13) a forward matrix $\varvec{B}\left( {\mathscr {L}}_n\right)$ was computed for all target points $\vec {r} \in {\mathscr {T}}$ and for all non-boundary vertices of our stream function discretization, with a level *n* mirroring. Coil wires must not cross the border of the mesh and hence for each target field vector $\varvec{b}_{\mathrm{target}}$, a regularized least squares solution of the stream function vector $\hat{\varvec{s}}$ was found from a pseudoinverse $\varvec{B}^{+}$:

22 $$\begin{aligned} \varvec{B}^{+}&= \alpha \left[ \alpha \varvec{B}^{\mathsf {T}} \left( {\mathscr {L}}_n\right) \varvec{B}\left( {\mathscr {L}}_n\right) +\lambda ^2 \varvec{I}\right] ^{-1} \varvec{B}^{\mathsf {T}} \left( {\mathscr {L}}_n\right) \end{aligned}$$

23 $$\begin{aligned} \hat{\varvec{s}}&= \varvec{B}^{+} \varvec{b}_{\mathrm{target}}\,, \end{aligned}$$

where $\alpha$ is a weighting parameter, $\lambda$ is a regularization parameter, $\varvec{I}$ is an identity matrix, with its diagonal length equal to the length of the non-boundary stream function vector, here 1537. The weighting parameter $\alpha$ was set such that

24 $$\begin{aligned} {\mathrm{trace}}\left( \varvec{B}^{\mathsf {T}}\left( {\mathscr {L}}_n\right) \cdot \alpha \cdot \varvec{B}\left( {\mathscr {L}}_n\right) \right) / {\mathrm{trace}}\left( \varvec{I}\right) = 1. \end{aligned}$$

**Figure 5:**
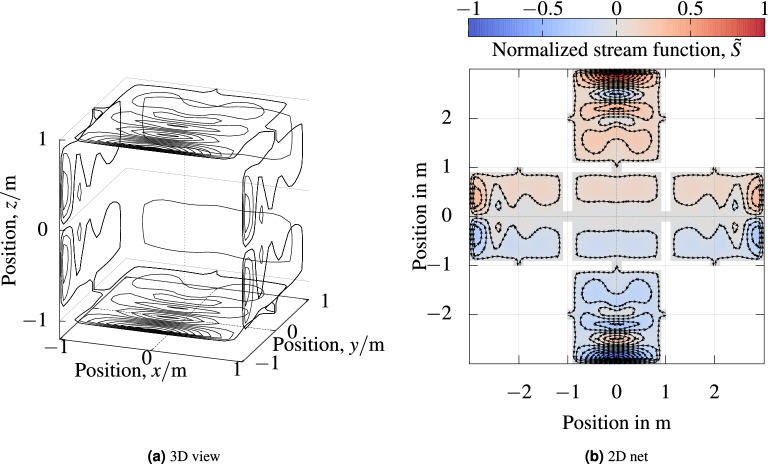
*Wire paths* (**a**) 3D view of stream function contour lines of our $C_{z,\,{\mathrm{hom}}}$ coil. In (**b**), a 2D net representation of the $C_{z,\,{\mathrm{hom}}}$ coil is depicted. Wire connections between stream function contours are not depicted. These connections can be implemented as twisted pair wires.

Boundary stream function values were set to zero. Contour lines of the so found stream function formed the wire paths on our meshes. The pseudoinverse in (23) was computed from a singular value decomposition of the forward matrix. For reproducibility the number of contour lines and regularization parameters of our coil optimization are listed in Table 2. Wire paths of one coil ($C_{z,\, {\mathrm{hom}}}$) are depicted in Fig. 5.

**Table 2:** Optimization parameters: number of contour lines and regularization parameter.

Coil	Number of contours	Regularization $\lambda$
$C_{x,\,{\mathrm{hom}}}$	20	0.023
$C_{y,\,{\mathrm{hom}}}$	20	0.003
$C_{z,\,{\mathrm{hom}}}$	20	0.004
$C_{x,\,\nabla _y}$	28	0.037
$C_{x,\,\nabla _x}$	28	0.006
$C_{z,\,\nabla _z}$	28	0.060
$C_{x,\,\nabla _z}$	20	0.001
$C_{z,\,\nabla _y}$	28	0.024

### Error estimates

The relative difference measure (RDM) measures differences in field topography^14^. It is defined as

25 $$\begin{aligned} RDM = \left\Vert \frac{\varvec{b}}{\left\Vert \varvec{b} \right\Vert } -\frac{\varvec{b}_{\mathrm{target}}}{\left\Vert \varvec{b}_{\mathrm{target}} \right\Vert } \right\Vert \in [0, 2]. \end{aligned}$$

As a second informative topography difference measure, which was also reported in^8,10^, the maximum relative difference was computed as

26 $$\begin{aligned} MRD = \max \limits _{n=1 \dots N} \left\{ \left|\frac{b_{n}}{\max \left\{ \varvec{b}_n \right\} } -\frac{b_{target\,n}}{\max \left\{ \varvec{b}_{\mathrm{target}} \right\} } \right|\right\} . \end{aligned}$$

To quantify field magnitudes of different levels of mirroring *i*, *j*, we computed the magnification (MAG) as

27 $$\begin{aligned} MAG = \frac{\left\Vert \varvec{b}\left( {\mathscr {L}}_i\right) \right\Vert }{\left\Vert \varvec{b}\left( {\mathscr {L}}_j\right) \right\Vert }. \end{aligned}$$

### Efficiency

Coil efficiency was computed for each coil as

28 $$\begin{aligned} EFF = \frac{\varvec{b}\left( I\right) \cdot \tilde{\varvec{b}}_{\mathrm{target}}}{I \cdot \tilde{\varvec{b}}_{\mathrm{target}} \cdot \tilde{\varvec{b}}_{\mathrm{target}}} \end{aligned}$$

where $\varvec{b}\left( I\right)$ is the magnetic field vector at the target points dependent on the current *I* in a coil and $\tilde{\varvec{b}}_{\mathrm{target}}$ is a unit-less target field vector derived from one right hand side of Eqs. (14) to (21). It is the averaged field or gradient at the target points per coil current.

## Results

**Figure 6:**
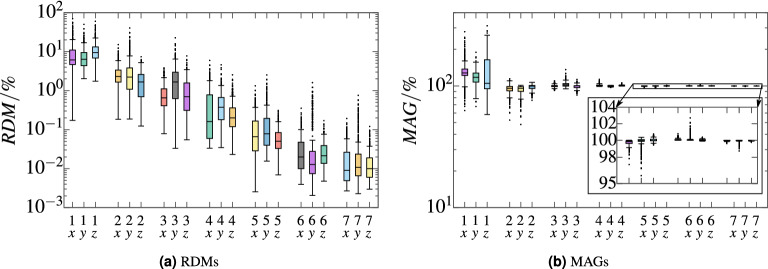
*Convergence of the method of images* RDMs (**a**) and MAGs (**b**) computed over columns of the forward matrix $\varvec{B}\left( {\mathscr {L}}_n\right)$ for increasing levels of mirroring *n*. The boxes are drawn around the region between the first and third quartiles, with a horizontal line at the median value. Whiskers extend from the box to the most distant values within 1.5 times the interquartile range. Outliers outside 1.5 times the interquartile range are marked with black dots.

**Figure 7:**
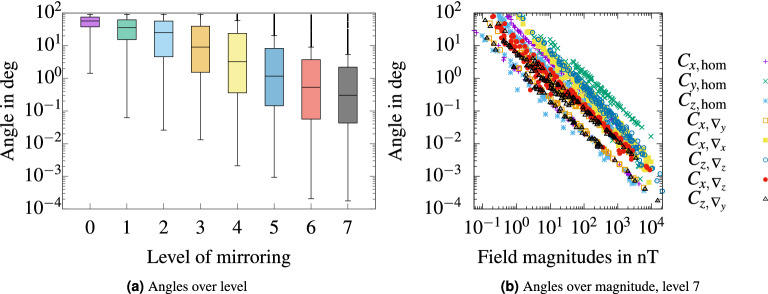
*Field angles to normal vectors of MSR walls* In (**a**), boxplots of field angles to normal vectors of the MSR walls are depicted for 600 boundary points over the level of mirroring *n*. The angles the of fields of all 8 coils are included in the boxplots. The boxes are drawn around the region between the first and third quartiles, with a horizontal line at the median value. Whiskers extend from the box to the most distant values within 1.5 times the interquartile range. Outliers outside 1.5 times the interquartile range are marked with black dots. In (**b**), the angles of the fields to the wall normals at the 600 points are depicted over the according field magnitudes for mirroring level 7 and for each coil seperately.

Convergence of the magnetic field at target points with increasing levels of mirroring is depicted in Fig. 6a,b. For each level of mirroring, the RDM and MAG was computed against the preceding level for each field component, *x*, *y*, *z*. Boxes labeled as $^k_a$, depict the $RDM^{k}_{n\,a}$ or $MAG^{k}_{n\,a}$ over all $n = 1 \dots N$ surface vertices, where *k* is the level of mirroring and *a* is one of the axes *x*, *y*, *z*. Formally these RDMs were computed as $RDM^{k}_{n\,a} = RDM\left( \varvec{b}^{k}_{n\,\varvec{a}}, \varvec{b}^{k-1}_{n\,\varvec{a}}\right)$, where $\varvec{b}^{k}_{n\,\varvec{a}} =\left( b_{n\,\#a}\left( {\mathscr {L}}_k\right) , b_{n\,\#a+3}\left( {\mathscr {L}}_k\right) \dots \right)$ is every third element of the *n*th row of $\varvec{B} \left( {\mathscr {L}}_k\right)$, $\#x = 1,\,\#y = 2,\,\#z=3$. Corresponding expressions apply for MAGs. For a level of mirroring of 7, the maximum RDM to the preceding level was 0.8% and according median values were $\approx$ 0.01%. These values represented the accuracy we were aiming for. The MAGs for a level of mirroring of 7 were between 98.8 and 100.0%. In contrast, single mirroring on each wall, level 1, compared to no mirroring at all resulted in a maximum RDM of 70% and a maximum MAG of 312%. Figure 7 demonstrates the reliability of the mirroring approach in terms of field boundary conditions. Therefore, the field vectors were evaluated at 100 points on each MSR wall. The evaluation points were placed at the 9th order Gauss-Legendre quadrature points in each wall.

**Figure 8:**
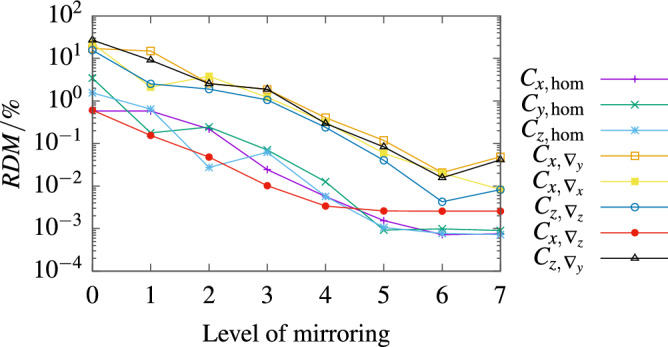
*Convergence of mirroring with BEM* RDMs of magnetic fields in the validation grid from the mirroring approach and BEM are depicted over the mirroring level. The RDM data is depicted for all eight coils.

The fields of our coils in the validation grid were compared against a boundary element method (BEM) solution. For the BEM computation, the boundary between air the inner $\mu$-metal sheet was triangulated with 14,506 triangles. The relative permeability of the $\mu$-metal was set to 80,000 and the Neumann boundary conditions were modeled with discontinuous constant shape functions on the triangles. In Fig. 8 a comparison of coil mirroring and BEM is depicted over the mirroring level.

Table 3 lists compensation errors, RDMs and maximum relative differences (MRDs), for our 8 coils in our validation target volume ${\mathscr {T}}_{\mathrm{val}}$ with diameter 0.6 m. RDMs and MAGs were computed over all target grid points and all spatial field components *x*, *y*, *z* for each coil. For a mirroring level of 7, RDMs were between 0.03 and 0.77%, MRDs between 0.05 and 1.10%. For a level of 7 the errors were small because the coils were optimized for that level, the RDMs and MRDs in the columns without mirroring revealed the effect of magnetic shielding on compensation performances.

**Table 3:** Compensation errors in ${\mathscr {T}}_{\mathrm{val}}$ (diameter 0.6 m), for simulations with mirroring (${\text {n}} = 7$) and without mirroring (${\text {n}} = 0$).

Coil	Error, $RDM/\%$ level $n = 7$	Error, $MRD/\%$ level $n = 7$	Error, $RDM/\%$ level $n = 0$	Error, $MRD/\%$ level $n = 0$
$C_{x,\,{\mathrm{hom}}}$	0.07	0.30	0.55	1.78
$C_{y,\,{\mathrm{hom}}}$	0.06	0.23	3.40	10.31
$C_{z,\,{\mathrm{hom}}}$	0.12	0.43	1.60	6.51
$C_{x,\,\nabla _y}$	0.48	0.88	17.30	18.84
$C_{x,\,\nabla _x}$	0.10	0.14	22.32	15.36
$C_{z,\,\nabla _z}$	0.17	0.44	15.56	11.89
$C_{x,\,\nabla _z}$	0.03	0.05	0.59	1.71
$C_{z,\,\nabla _y}$	0.77	1.10	27.30	31.06

In Table 4 the efficiency and wire length are listed for each coil. The efficiency is the average field or gradient in the target volume ${\mathscr {T}}_{\mathrm{val}}$ per coil current. There were correlations of 99% and 91% between efficiency and wire length for homogeneous and gradient coils, respectively. After correcting for direct proportionality between efficiency and wire length, gradient coils optimized for a gradient on the *y* axis were less efficient compared to other gradient coils. An explanation for this might be that, to the *y* axis, only one coil surface side was perpendicular whereas to the *x* and *z* axes, two sides were perpendicular.

**Table 4:** Coil parameters: efficiency and wire length.

Coil	Efficiency, *EFF*	Wire length, $\ell /{\mathrm{m}}$
$C_{x,\,{\mathrm{hom}}}$	2.1 $\upmu {\text {T}}/{\text {A}}$	154
$C_{y,\,{\mathrm{hom}}}$	5.0 $\upmu {\text {T}}/{\text {A}}$	436
$C_{z,\,{\mathrm{hom}}}$	1.3 $\upmu {\text {T}}/{\text {A}}$	129
$C_{x,\,\nabla _y}$	2.9 $\upmu {\text {T}}/({\text {m}}\,{\text {A}})$	190
$C_{x,\,\nabla _x}$	10.5 $\upmu {\text {T}}/({\text {m}}\,{\text {A}})$	382
$C_{z,\,\nabla _z}$	14.0 $\upmu {\text {T}}/({\text {m}}\,{\text {A}})$	353
$C_{x,\,\nabla _z}$	4.7 $\upmu {\text {T}}/({\text {m}}\,{\text {A}})$	273
$C_{z,\,\nabla _y}$	2.7 $\upmu {\text {T}}/({\text {m}}\,{\text {A}})$	184

## Discussion

We developed a framework to design compensation coils of arbitrary shape with quadrilateral meshes of coil surfaces. In this framework, shielding effects are incorporated by a method of images in coil optimization. Our design is based on a cuboid surface with 5 faces and a side length of approximately 2 m. However, our design methods can be applied to bi-planar coils or any wire distributions constrained to surfaces. The maximum MRD in ${\mathscr {T}}_{\mathrm{val}}$ was 1%, which matches our compensation error goal.

Our simulations of shielding effects by mirroring of currents converged with an increasing mirroring level. Fields in our target grid resulting from different levels of mirroring were compared. Between mirroring levels 6 and 7, their RDMs were clearly below our compensation error goal of 1%, with upper quartiles below 0.1%. The boundary conditions on the boundary between air and $\mu$-metal in our MSR require almost perpendicular magnetic field vectors to the boundary surface. This condition was evaluated for 600 points on the boundary surface. The deviation from this boundary condition was reduced by an increasing mirroring level. For a mirroring level of 7, it was found that this condition was well fulfilled for field vectors with large magnitudes. For small magnitudes, the angle is not well defined numerically and the effect of deviations from the boundary condition on the target field is small. The results of the mirroring approach in our target volume were further confirmed by a corresponding BEM computation. With increasing mirroring level, the RDMs of fields from the mirroring approach and BEM in the target grid converged to values below 0.1%. A limitation of this method of images is that it cannot account for the second $\mu$-metal sheets, which are at different distances from a point inside the MSR. It also does not account for the thickness of $\mu$-metal sheets. A theoretical requirement of this method is that the shielding surface is closed, which was not entirely the case in practice. There were several holes and discontinuities in the $\mu$-metal sheets of our MSR, for example holes for cables, ventilation, video projector light, a helium tube, and discontinuities in the door frame. Effects of these limitations and unmet requirements are a study on their own and are not part of this paper. To account for the mentioned conditions, other numerical methods like boundary element or finite element methods can be applied and validated against empirical data.

The difference between our shielding effect simulations and the methods described in^10^ is that we incorporated these simulations in coil optimizations. That is, our coils were optimized for a specific magnetically shielded environment, our MSR. Reference^10^ reported simulated and measured shielding effects on coil performances whereas their coils were optimized for an unshielded environment. To simulate shielding effects on their coil performances, they used a method of images with a mirroring level of 3. Their MSR is of the same dimensions as ours, in fact it is the same model, produced by the same company. They compared shielding simulations with empirically measured data inside their MSR and found good agreement between simulation and measurement. Once the coils are build and in place, we will also compare our simulations with measured data.

Compared to^10^, we evaluated the effect of shielding on our coil performance in reverse. That is, for our coils, which were optimized for a position inside our MSR, compensation errors were computed for an unshielded environment, as listed in the last two columns in Table 3. This revealed the importance of incorporating shielding effects in coil designs. These error values are comparable to the reverse findings of^10^ with coils optimized for an unshielded environment and error computation with shielding.

Recently, an open source software was released that is able to model arbitrary coil surface geometries and incorporate magnetic shielding in coil designs^15^. In this paper, we describe and evaluate specific coils whereas^15^ implemented a generic tool for coil designs. In retrospect, the methods of^15^ could be applied to our modeling problem but were unavailable to us at that time. We expect that it is straightforward to reproduce our coils and results from our description by using this software and to end up with similar coils. However, this has not been tested yet. In contrast to^15^, we use quadrilaterals instead of triangles for surface discretization and we model magnetic shielding by a method of images instead of using collocation points for a scalar potential close to the shielding surface. Quadrilaterals are a natural discretization choice for our cuboid geometry. Compared to a scalar potential method, a method of images does not have to deal with singularities at the shielding surface.

## Conclusion

We proposed compensation coil designs that achieved higher accuracy in a larger target volume compared to reported results for similar dimensions^8,10^. Improved compensation compared to^8,10^ came at the price of a more complex construction. We will compare our simulations with empirically measured fields inside our MSR in the future.

## Supplementary Information

Supplementary Information.
