# Supplementary material for: Magnetic field compensation coil design for magnetoencephalography
Source: Sci Rep. 2021 Nov 22;11:22650. doi: 10.1038/s41598-021-01894-z (PMC8608906; doi:10.1038/s41598-021-01894-z)
Supplement: Supplementary file 1 — Supplementary Information. [file 41598_2021_1894_MOESM1_ESM.pdf]

Magnetic field compensation coil design for magnetoencephalography

Supplementary material

Hermann Kutschka Christian F. Doeller Jens Haueisen
Burkhard Maess

Model files of mirroring examples of simple meshes as in Fig. S1a, Fig. S1b, Fig. S1c can be found at `https://compensation-coil-design.pages.gwdg.de/supplementary-material`. The purpose of these models and figures was to demonstrate the meaning of mirroring levels.

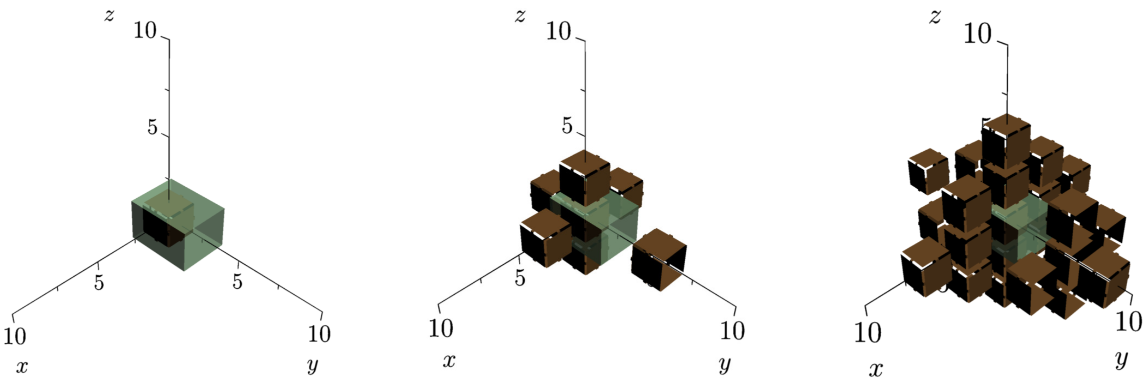

(a) Mirroring level 0 (b) Mirroring level 1 (c) Mirroring level 2

Figure S1: The walls of the magnetically shielded room are indicated in green color. The surface meshes at the wooden boards inside the magnetically shielded room are indicated in brown color. The mirrored surface meshes outside the magnetically shielded room are indicated in brown color for different mirroring levels in (a), (b), (c).

In Fig. S2 to Fig. S9, the wire paths are depicted in 3D views and 2D representations along with current flow directions and normalized stream function maps. The data and code to produce these figures can be found at
`https://compensation-coil-design.pages.gwdg.de/supplementary-material`.

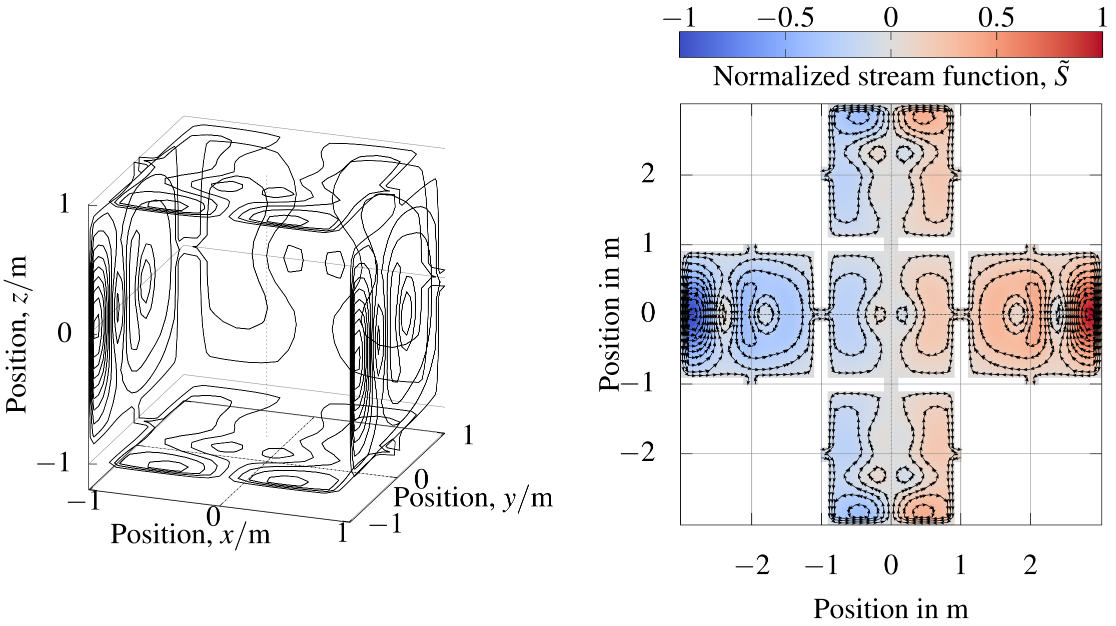

(a) 3D view

(b) 2D net

Figure S2: **Wire paths** (a) 3D view of stream function contour lines of our $C_{x,\,\mathrm{hom}}$ coil. In (b), a 2D net representation of the $C_{x,\,\mathrm{hom}}$ coil is depicted.

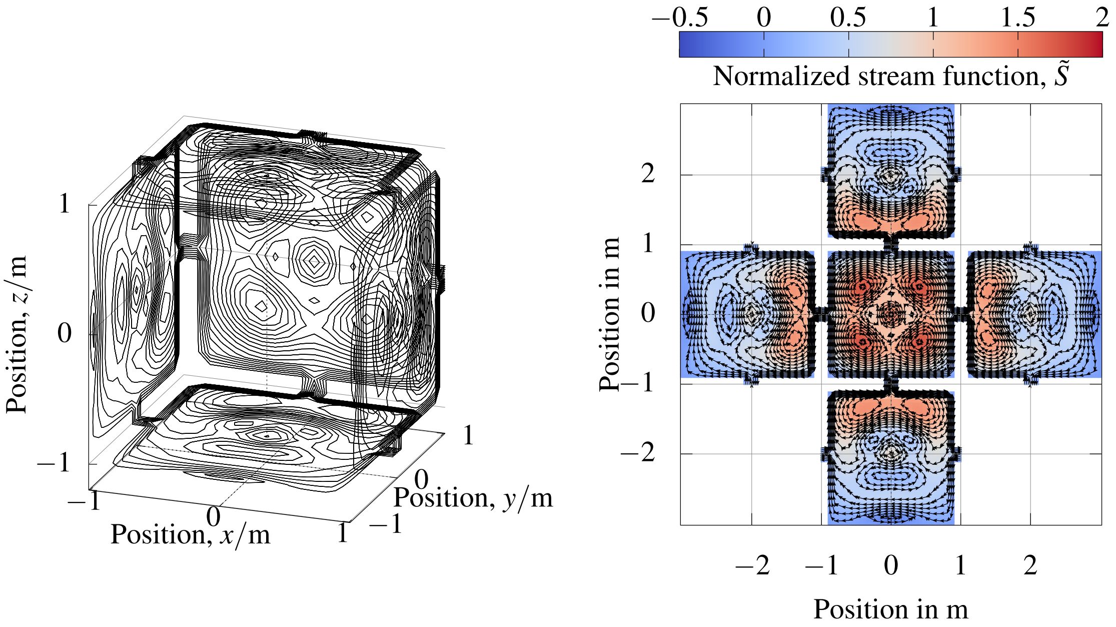

(a) a

(b) b

Figure S3: **Wire paths** (a) 3D view of stream function contour lines of our $C_{y,\,\mathrm{hom}}$ coil. In (b), a 2D net representation of the $C_{y,\,\mathrm{hom}}$ coil is depicted.

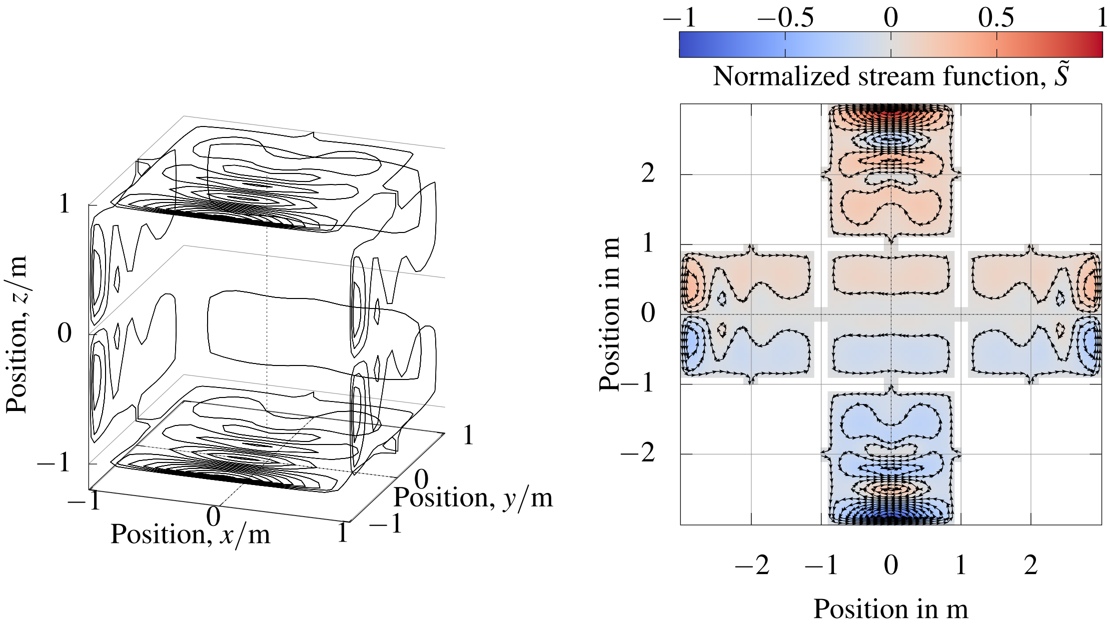

(a) 3D view (b) 2D net

Figure S4: **Wire paths** (a) 3D view of stream function contour lines of our $C_{z,\,\mathrm{hom}}$ coil. In (b), a 2D net representation of the $C_{z,\,\mathrm{hom}}$ coil is depicted.

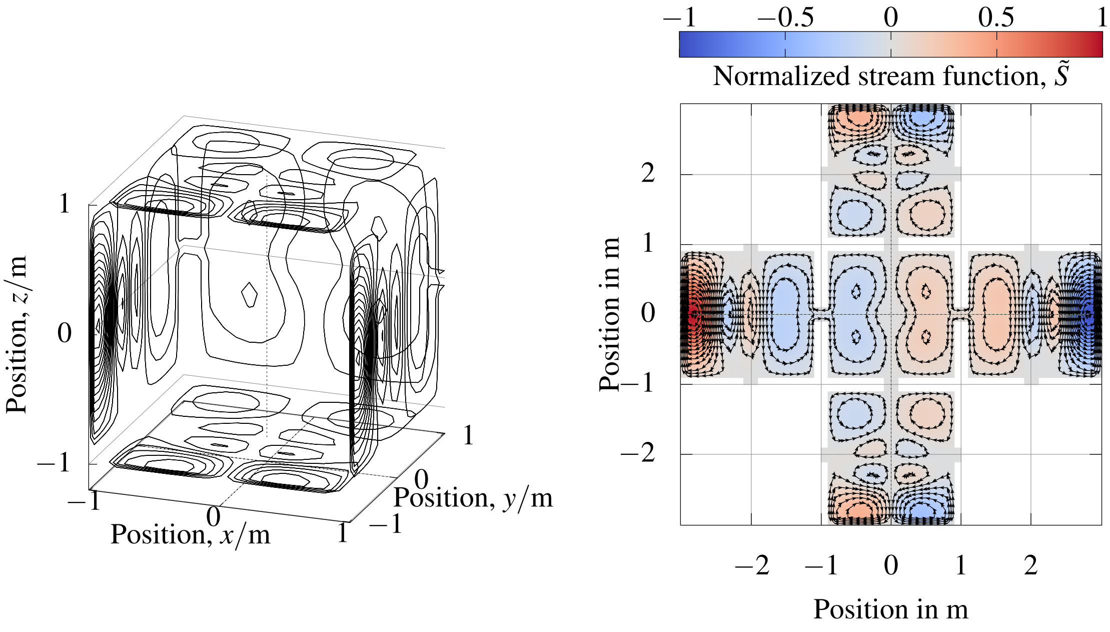

(a) 3D view (b) 2D net

Figure S5: **Wire paths** (a) 3D view of stream function contour lines of our C_{x,∇_y} coil. In (b), a 2D net representation of the C_{x,∇_y} coil is depicted.

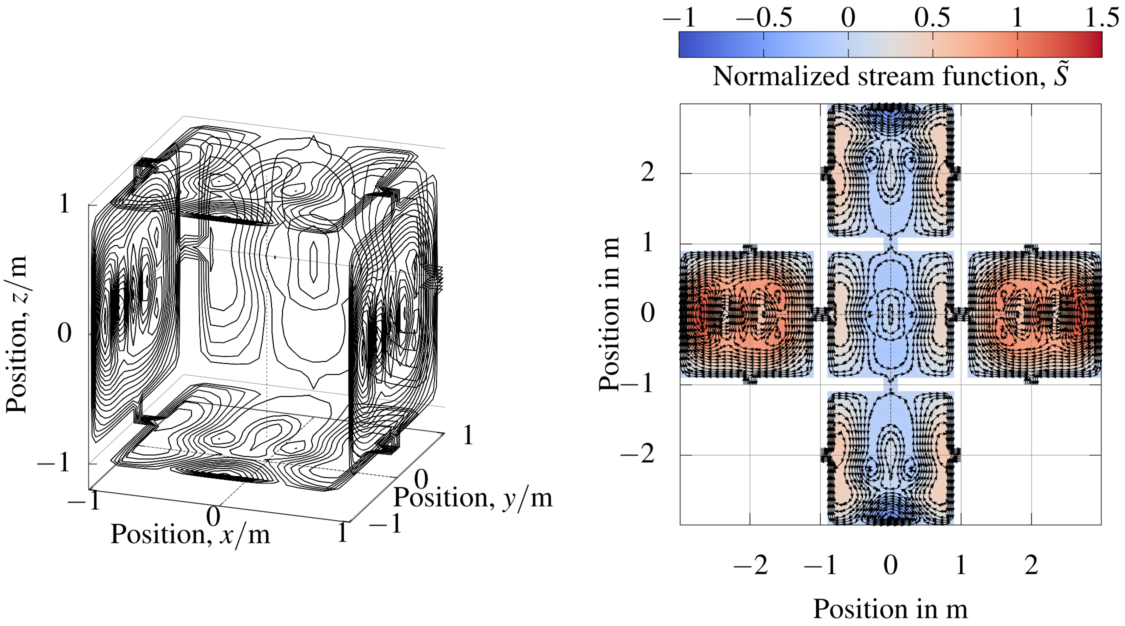

(a) 3D view (b) 2D net

Figure S6: **Wire paths** (a) 3D view of stream function contour lines of our C_{x,∇_x} coil. In (b), a 2D net representation of the C_{x,∇_x} coil is depicted.

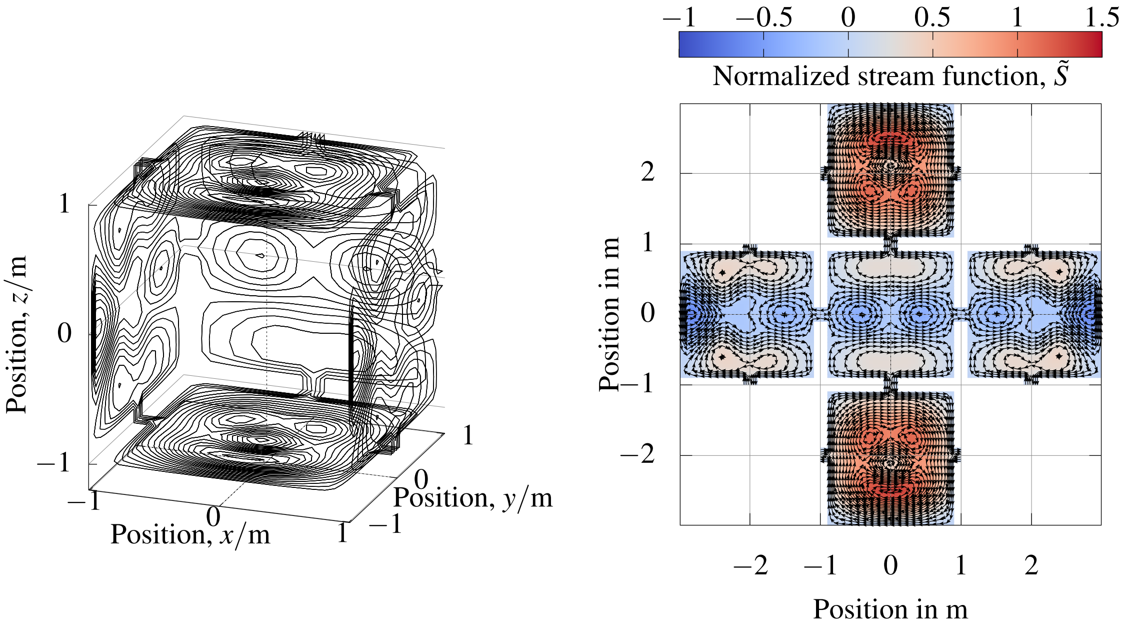

(a) 3D view (b) 2D net

Figure S7: **Wire paths** (a) 3D view of stream function contour lines of our C_{z,∇_z} coil. In (b), a 2D net representation of the C_{z,∇_z} coil is depicted.

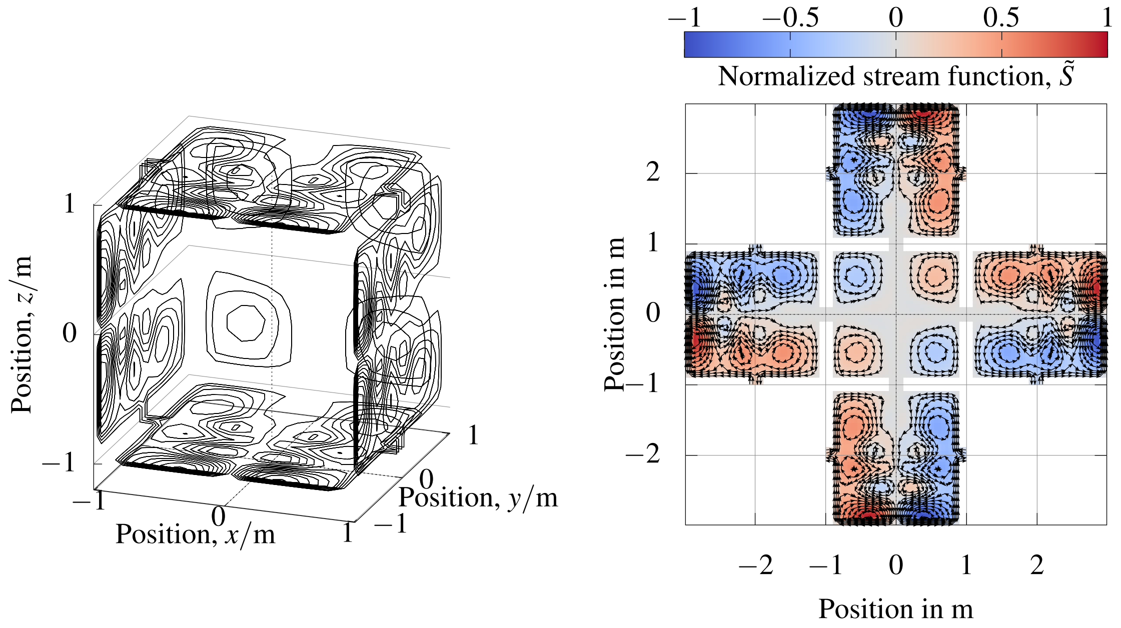

(a) 3D view (b) 2D net

Figure S8: **Wire paths** (a) 3D view of stream function contour lines of our C_{x,∇_z} coil. In (b), a 2D net representation of the C_{x,∇_z} coil is depicted.

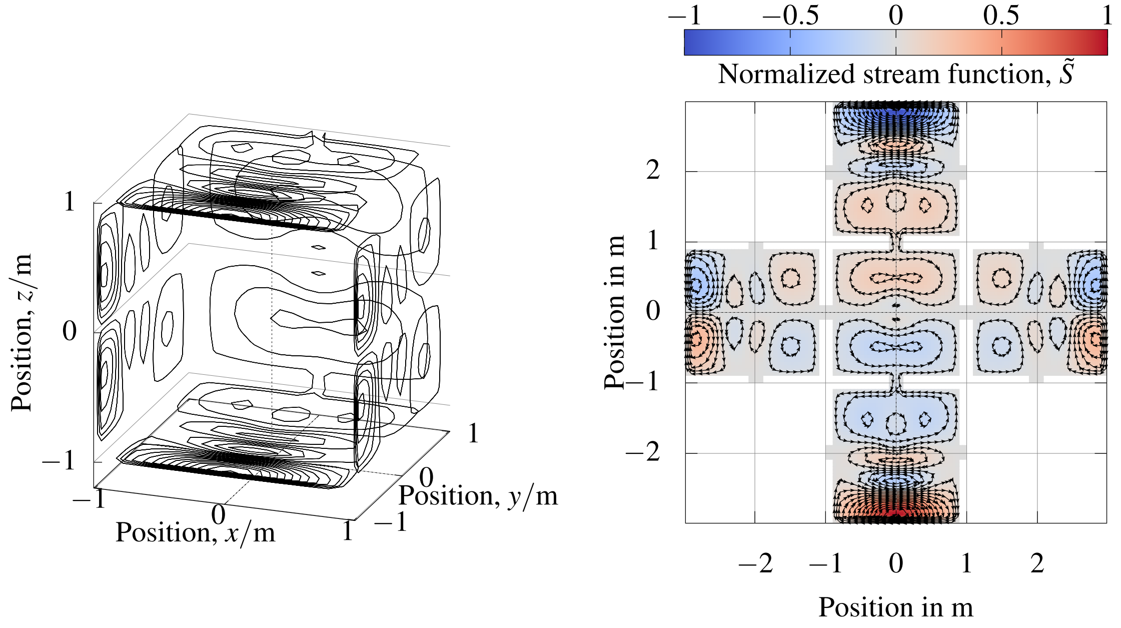

(a) 3D view (b) 2D net

Figure S9: **Wire paths** (a) 3D view of stream function contour lines of our C_{z,∇_y} coil. In (b), a 2D net representation of the C_{z,∇_y} coil is depicted.
